# Rapidly progressive renal failure due to tubulointerstitial infiltration of peripheral T-cell lymphoma, not otherwise specified accompanied by uveitis: a case report

**DOI:** 10.1186/s12882-018-1125-9

**Published:** 2018-11-08

**Authors:** Ken Matsuda, Hirotaka Fukami, Ayako Saito, Hiroyuki Sato, Satoshi Aoki, Yoichi Takeuchi, Shinji Nakajima, Tasuku Nagasawa

**Affiliations:** 1 Department of Nephrology, Japanese Red Cross Ishinomaki Hospital, 71 Nishimichisita Hebita Ishinomaki, Miyagi, 986-8522 Japan; 2 Department of Hematology, Japanese Red Cross Ishinomaki Hospital, Miyagi, Japan

**Keywords:** Peripheral T-cell lymphoma not otherwise specified, Tubulointerstitial nephritis, Rapidly progressive renal failure, Splenohepatomegaly, Uveitis masquerade syndrome, Pulse steroid therapy

## Abstract

**Background:**

Rapid decline in renal dysfunction due to primary renal lymphoma, or secondary renal lymphoma by infiltration from a primary origin, is extremely rare. There are notably few reports indicating infiltration of T-cell lymphoma into the kidney.

**Case presentation:**

A 61-year-old woman with a sudden body rash and liver dysfunction was brought to our hospital presenting with a dull headache and blurred vision. Laboratory tests revealed rapidly progressive renal failure. Histological examination of the kidney and skin indicated infiltration of peripheral T-cell lymphoma, not otherwise specified (PTCL-NOS). Infiltration of PTCL-NOS to the liver and spleen, and presence of Uveitis masquerade syndrome were suspected. Imaging showed that the lesion was limited to extralymphatic organs. Renal function was improved with administration of steroids, including pulse steroid therapy, before administering cyclophosphamide, doxorubicin, vincristine, and prednisolone (CHOP) therapy.

**Conclusions:**

This is the first reported case of rapidly progressive renal failure caused by perivascular tubulointerstitial nephritis with the direct invasion of PTCL-NOS. In our case, a single steroid dose showed dramatic results with respect to renal symptoms.

## Background

Malignant lymphoma is largely classified into Hodgkin and non-Hodgkin’s lymphoma. In Japan, Hodgkin’s lymphoma comprises only 5% of all malignant lymphoma cases. Cases of non-Hodgkin’s lymphoma are classified as B-cell lymphoma or T-cell/NK-cell lymphoma.

An earlier report revealed that in a series of 322 autopsies of patients who had died of malignant lymphoma, the kidneys were involved in 121 (37.6%) patients [1]. Involvement of the kidneys in lymphoma is common. However, rapid decline in renal dysfunction due to primary renal lymphoma, or secondary renal lymphoma by infiltration from a primary origin, is extremely rare. Reports of such cases in literature are sparse, because there is no lymph tissue in the renal parenchyma [2]. Infiltration of malignant lymphoma into the kidney is usually caused by B-cell lymphoma, and few reports of infiltration of T-cell lymphoma exist. Additionally, we did not find rapid progressive renal failure caused by peripheral T-cell lymphoma not otherwise specified (PTCL-NOS) in the published literature.

Peripheral T-cell lymphoma (PTCL) is a general term for lymphoma that originates in the T cells and migrates to peripheral organs after differentiated maturity in the thymus gland. There are three main types of PTCL: PTCL not otherwise specified (PTCL-NOS), angioimmunoblastic T-cell lymphoma (AITL) which is a nodal type of “aggressive lymphoma”, and anaplastic large-cell lymphoma (ALCL) [3, 4].

Based on the WHO classification, adult T-cell leukemia lymphoma (ATLL) comprises approximately 25% of all lymphoid tumors in Japan. A relatively high number of ATLL cases are caused by human T-lymphotropic virus type I (HTLV-I). Among these, PTCL-NOS comprises 6.7%, AITL comprises 2.4%, and ALCL comprises 1.5%. B-cell lymphoma has various established standard treatments, including anti-CD20 monoclonal antibody treatment. However, for PTCL, there are few established strategies for effective treatment and the prognosis is poor. Cyclophosphamide, doxorubicin, vincristine, and prednisolone (CHOP) therapy is the preferred frontline treatment for PTCL. A systematic review, comprising 2815 PTCL patients, reported that the 5-year overall survival rate was 38.5% with CHOP or CHOP-like regimens [5].

We report a case of PTCL-NOS with multiple findings not only in the kidney, but also in the eye, liver, and dermis that progressed rapidly. We succeeded in improving renal and liver function, and preventing blindness, with rapid steroid administration before introduction of CHOP therapy.

## Case presentation

**Fig. 1:**
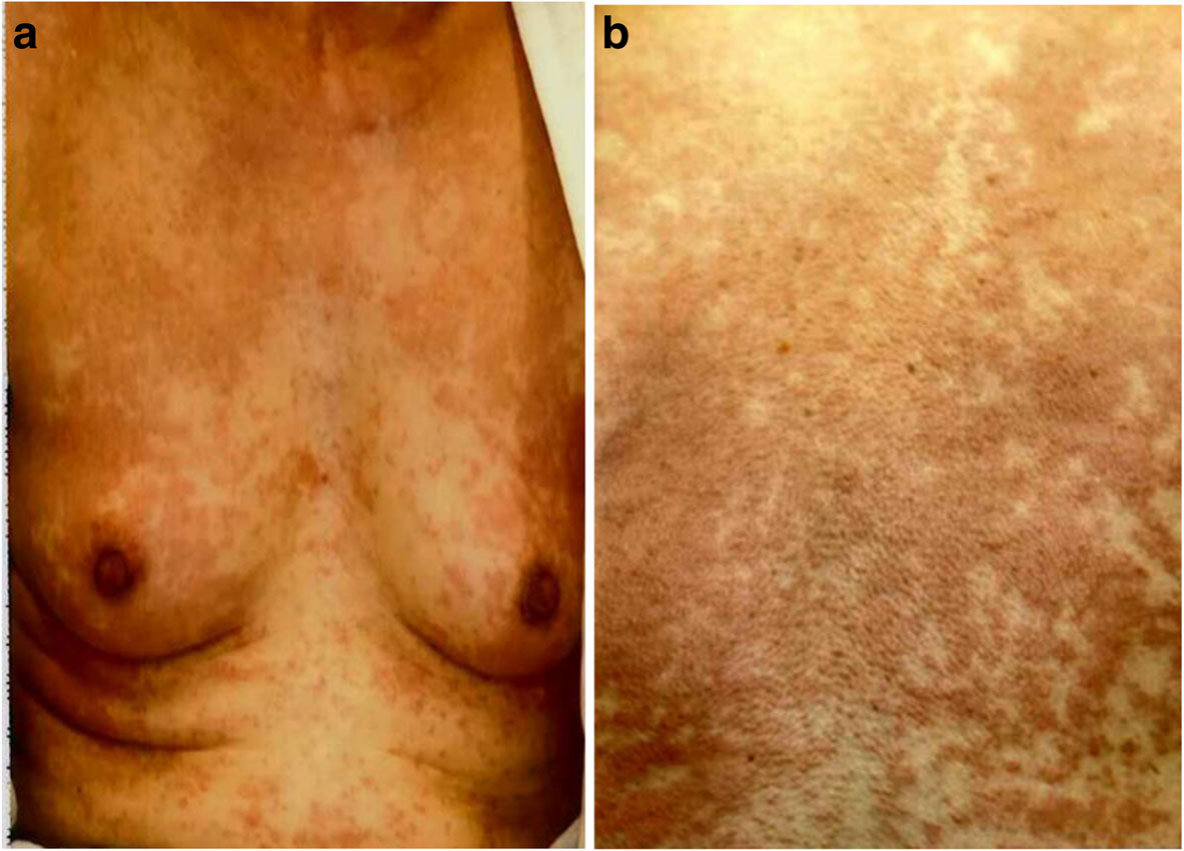
Physical findings on admission. **a** Rash on the chest, (**b**) Rash on the back

We present the case of a 61-year-old woman with a history of total hysterectomy owing to a uterine fibroid at the age of 35 years. Nine days before admission to our hospital, she developed an itchy rash covering the whole body. Seven days before admission to our hospital, she visited a dermatologist who prescribed oral and topical medicines; however, there was no improvement. Three days before admission to our hospital, she visited a physician for general malaise and loss of appetite. Liver function disorder was detected by blood tests (AST (aspartate aminotransferase), 165 U/L; ALT (alanine transaminase), 291 U/L; ALP (alkaline phosphatase), 840 U/L; γ-GTP (γ-glutamyl transpeptidase), 373 U/L) and thickening of the gallbladder wall was seen on abdominal echo imaging. She was referred to our department of gastroenterological medicine. However, on the day of her visit to our hospital, she experienced a dull headache and blurred vision on attempting to get out of bed. The itching increased and she was brought to our emergency outpatient department. On admission, her clinical parameters were as follows: height, 162 cm; weight, 46.5 kg; JCS (Japan Coma Scale), 0; temperature, 36.6 °C; blood pressure, 126/82 mmHg; pulse, 77 bpm; and peripheral capillary oxygen saturation (SpO_2_), 96% (room air). Conjunctival congestion and jaundice were present, and breathing sounds were normal. Several erythemas (millimeter size), itching sensations on the face, body, and upper and lower extremities, partially fused wheals, and small papules were also observed (Fig. 1a, b). There was no dryness of the mouth, pedal edema, decreased body weight, purpura, superficial lymph nodes, or nocturnal sweating. Laboratory results are shown in the Table 1. The results (creatinine (Cr) 3.08 mg/dL) indicated rapid decline in renal function compared to the tests conducted 3 days prior to admission (Cr 0.74 mg/dL). In addition, liver function tests were also abnormal. The patient was referred to the nephrology department and admitted to our hospital for examination and treatment.

**Table 1:** Laboratory results on admission

WBC 9.2×10^3^/μl	TP 5.9 g/dl	BUN 48.2 mg/dl	HCV (-)	Urine specific gravity 1.015
Seg 59 %	Alb 2.8 g/dl	Cr 3.08 mg/dl	HBsAg (-)	Urine pH 5.5
Lymph 12 %	T-bil 3.3 mg/dl	UA 7.9 mg/dl	ATLA (-)	Urinary sugar (-)
Mono 15 %	AST 71 IU	Na 132 mEq/l	TPHA (-)	Urine RBC 1-4/HPF
Eos 0 %	ALT 172 IU	K 4.2 mEq/l	RPR (-)	Uric protein 1.1 g/gCre
Baso 0 %	ALP 1021 IU	Cl 96 mEq/l	HIV (-)	NAG 13.8 IU/l
AT-Ly 1.5 %	γGTP 400 IU	Ca 8.7 mg/l		Urine β2MG 3090 μg/l
MMy 1.0 %	LDH 320 IU	P 3.3 mg/dl		
RBC 455×10^4^/μl	CRP 3.76 mg/dl	β2MG 3.1 mg/l		
Hb 13.3 g/dl	Ferritin 198 ng/ml			
MCV 86.6 fl	IL-2R 7250 U/ml			
Plt 31.4×10^4^/μl				

*WBC* (white blood cells); Seg (segmented neutrophils), *Lymph* (lymphocytes), *Mono* (monocytes), *Eos* (eosinophils), *Baso* (basophils), *AT-Ly* (atypical lymphocytes), *MMy* (multiple myeloma cells), *RBC* (red blood cells), *Hb* (hemoglobin), *Plt* (platelets), *MCV* (mean corpuscular volume), *TP* (total protein), *Alb* (albumin), *T-bil* (total bilirubin), *AST* (aspartate aminotransferase), *ALT* (alanine transaminase), *ALP* (alkaline phosphatase), *γ-GTP* (γ-glutamyl transpeptidase, *LDH* (lactate dehydrogenase), *CRP* (C-reactive protein), *IL-2R* (interleukin-2 receptor), *Na* (sodium), *K* (potassium), *Cl* (chloride), *Ca* (calcium), *P* (phosphate), *BUN* (blood urea nitrogen), *Cr* (creatinine), *UA* (uric acid), *HCV* (hepatitis C virus), *HBsAg* (hepatitis B surface antigen), *ATLA* (human T-cell leukemia virus typeΙantigen), *TPHA* (treponema pallidum hemagglutination test), *RPR* (rapid plasma reagin), *HIV* (human immunodeficiency virus), *β2MG* (beta 2 microglobulin), *NAG* (N-acetyl-β-D-glucosaminidase)

**Fig. 2:**
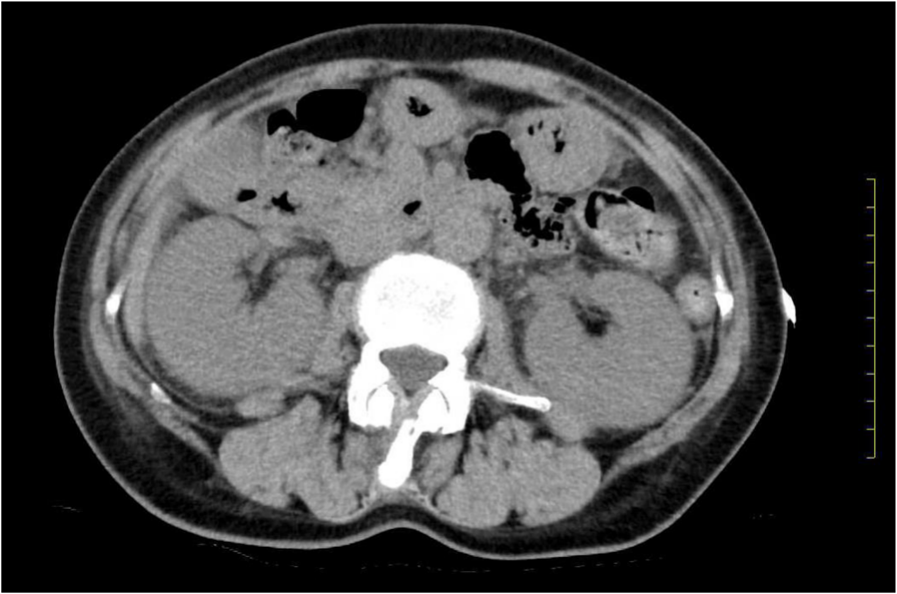
Abdominal computed tomography (CT) showing slight enlargement of both kidneys (right, 12 × 7 cm; left, 11 × 6 cm)

**Fig. 3:**
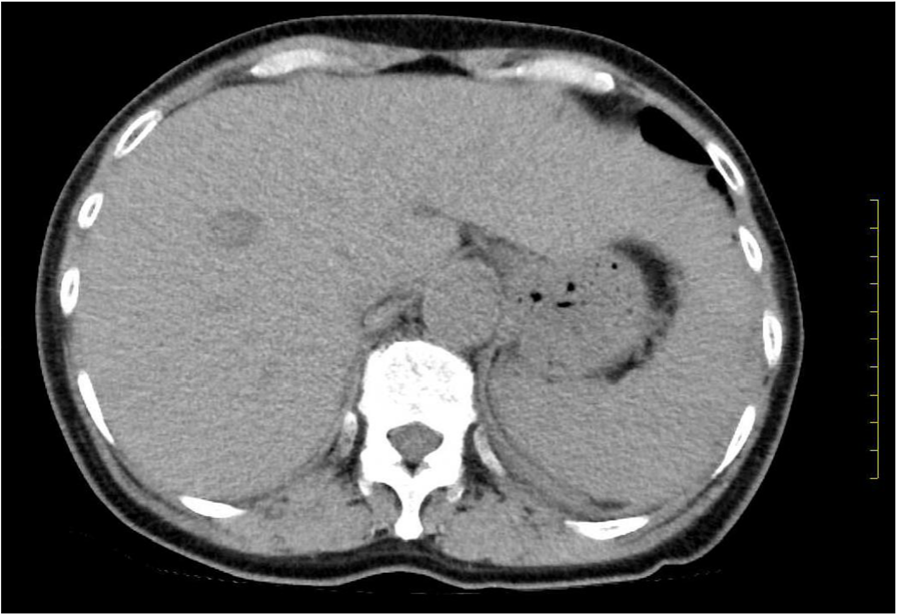
Abdominal computed tomography (CT) showing splenohepatomegaly (major axis of the spleen, 10.5 cm; size of the right hepatic lobe, 15.0 cm; size of the left hepatic left lobe, 12.0 cm)

**Fig. 4:**
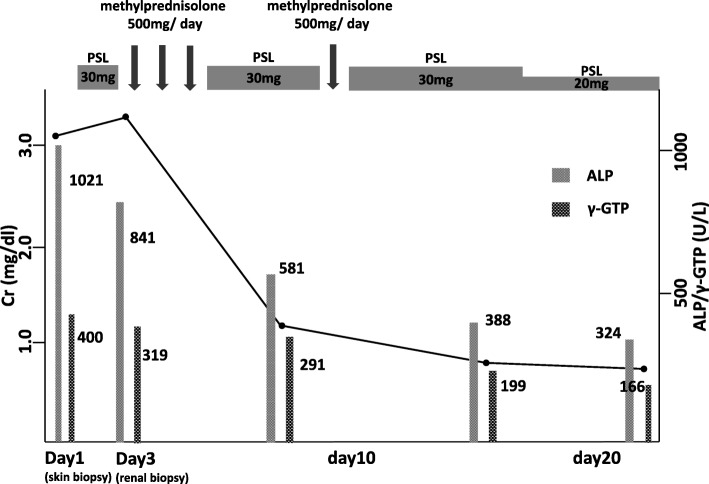
Clinical course of this case ALP (alkaline phosphatase); Cr (creatinine); γ-GTP (γ-glutamyl transpeptidase); PSL (prednisolone)

**Fig. 5:**
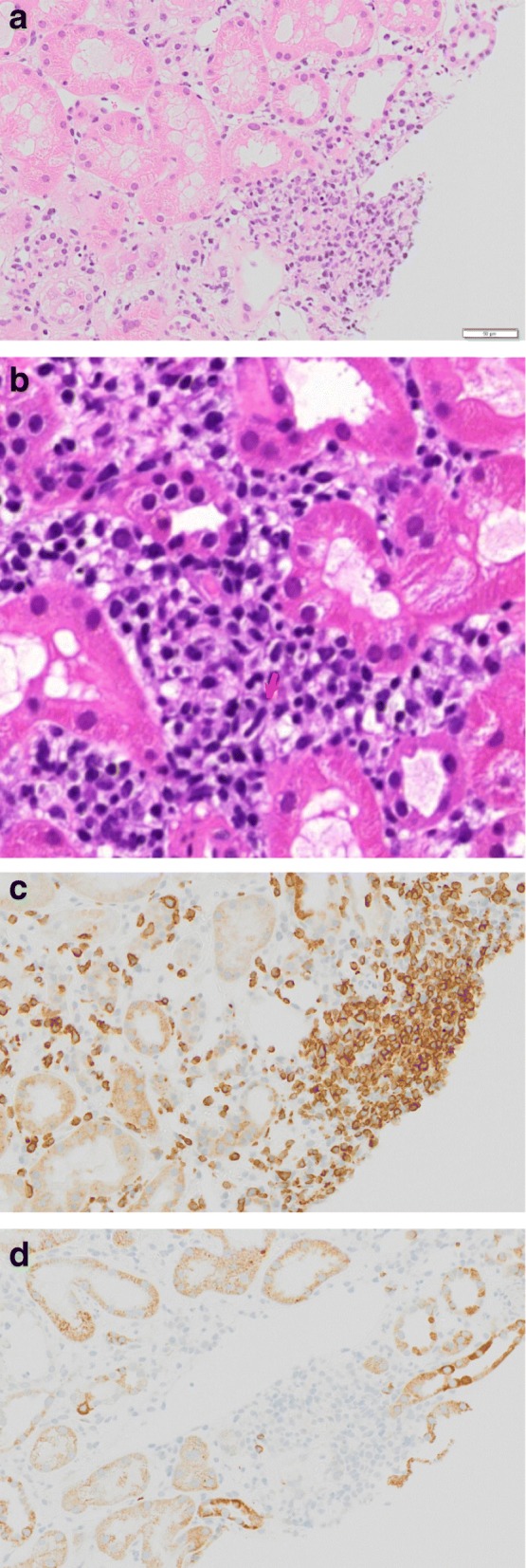
Renal histopathological findings. **a**, **b** Hematoxylin-eosin staining showing tubulointerstitial infiltration of atypical lymphocytes. **c** Immunostaining showing a high degree of CD3 expression in the tubule interstitium. **d** Immunostaining did not show CD20 expression in the tubule interstitium

**Fig. 6:**
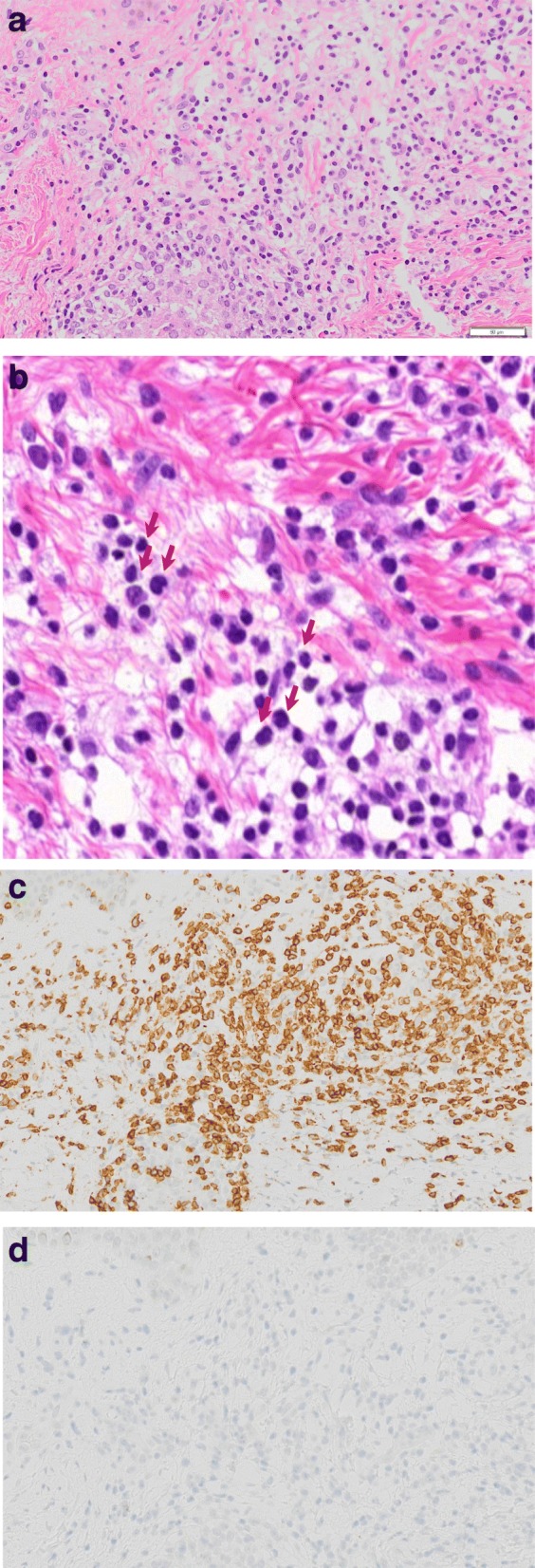
Dermat histopathological findings. **a**, **b** Hematoxylin-eosin staining showing subcutaneous tissue infiltration of atypical lymphocytes. **c** Immunostaining showing a high degree of CD3 expression in the subcutaneous tissue. **d** Immunostaining did not show CD20 expression in the subcutaneous tissue

Abdominal computed tomography (CT) showed slight enlargement of both kidneys (right, 12 × 7 cm; left, 11 × 6 cm) (Fig. 2). Hepatitis virus antigen/antibody tests were negative on admission and there was no history of drinking; however, hepatobiliary enzymes were elevated. In addition, abdominal CT showed splenohepatomegaly (Fig. 3). For the systemic rash, the patient was referred to the dermatology department on the day of admission, and a skin biopsy was performed. The rash was suspected to be an adverse effect of a drug; therefore, use of the previously prescribed drug was discontinued. The patient was also referred to the ophthalmology department for her blurred vision. Cataracts and uveitis were observed, along with increased intraocular pressure (IOP) (left IOP, 14 mmHg; right IOP, 13 mmHg). Abdominal CT did not reveal obstruction of the urinary tract, thus ruling out postrenal failure. Assuming the possibility of a prerenal failure, we administered extracellular fluid to maintain the hemodynamics. However, there was no improvement in renal function. We then suspected rapidly progressive renal failure with renal parenchyma involvement, or interstitial failure. Among the causes of rapidly progressive renal failure, we suspected nephrotoxic medications or glomerulonephritis due to membrane-type lupus nephritis or renal lymphoma. During hospitalization, her IOP further increased (left IOP, 35 mmHg; right IOP, 37 mmHg), for which various eye drops (steroids, prostaglandin-related drugs, beta-blocking drugs, adrenergic alpha 2 receptor agonists, carbonic anhydrase inhibitors, rho kinase inhibitors) were administered. However, there was no improvement. We administered oral steroids (prednisolone 30 mg/day) to prevent blindness and protect the kidneys. An improvement in the eye symptoms was detected. On day 3 of hospitalization, we performed a renal biopsy to determine the cause of rapidly progressive renal failure. In addition, after renal biopsy, we administered pulse steroid therapy (methylprednisolone 500 mg/day for 3 days) to protect the kidneys and further improve the eye symptoms (Fig. 4). The response to pulse steroid therapy was good and renal function gradually improved (day 3 of hospitalization, Cr 3.22 mg/dL; day 5, Cr 2.06 mg/dL; day 8, Cr 1.13 mg/dL) (Fig. 4). One complete course of pulse steroid therapy was administered and the dose of prednisolone was decreased to 20 mg/day from day 18. Elevated hepatobiliary enzymes gradually improved with steroids (Fig. 4). The systemic rash and itching sensation began to dissipate, although pigmentation was still visible. Her vision improved and IOP decreased, thus blindness was prevented. On day 22, a diagnosis of tubulointerstitial nephritis due to tubulointerstitial infiltration of PTCL-NOS was made, based on the results of renal biopsy (hematoxylin-eosin staining showed the presence of atypical lymphocytes; immunostaining showed that CD2, CD3, and CD4 were positive and CD5, CD7, CD8, and CD20 were negative) (Fig. 5a, b, c, d) and a Ki-67 score of approximately 80%. We also diagnosed subcutaneous tissue infiltration of PTCL-NOS, based on the results of skin biopsy (hematoxylin-eosin staining showed the presence of atypical lymphocytes; immunostaining showed that CD2, CD3, and CD4 were positive and CD5, CD7, CD8, and CD20 were negative) (Fig. 6a, b, c, d) and a Ki-67 score of approximately 80%. We performed flow cytometric analysis of the kidney and skin tissue, which showed similar results. We performed Southern blot analysis on kidney and skin tissue, but we could not obtain the result because of small amount of DNA. A presumptive diagnosis of PTCL-NOS to the liver and spleen and existence of Uveitis masquerade syndrome [6] due to PTCL-NOS was made based on the clinical course. Since lymph node lesions were not seen on imaging, we assumed that the lesions were limited to extralymphatic organs. In addition, we performed a spinal fluid test and found an atypical lymphocyte count of 5%. These atypical lymphocytes showed the same findings on flow cytometric analysis as those in the kidney and skin. The patient was referred to the hematology department and initial CHOP therapy was administered on day 23 of hospitalization.

For 8 months after admission, seven courses of CHOP therapy (Vincristine 1.4 mg/m^2^, Doxorubicin 50 mg/m^2^, Cyclophosphamide 750 mg/m^2^, Prednisolone 100 mg/day day1-day5) were administered and positron emission tomography/CT was performed. No enhanced uptake of FDG (fluorodeoxyglucose) was seen in any principal organs or lymph nodes, indicating complete remission. There was no significant change in the size of the liver; however, a decrease in the size of the spleen and both kidneys was seen.

## Discussion and conclusions

In this case, biopsy enabled us to confirm the infiltration of PTCL-NOS into renal and skin tissues. We diagnosed the case as PTCL-NOS, WHO classification of stage IV, based on pathological findings. PTCL-NOS is the commonest sub-type of PTCL. The pathogenesis remains unknown and no risk factors have been clearly identified. Wallett et al. reported that most PTCL-NOS cases present with late stage nodal disease, with extranodal involvement seen in two-thirds of cases [7, 8]. Skin and subcutaneous tissues are involved in more than 20% of the cases, which might appear as a primary cutaneous disease (pcPTCL-NOS) or as a part of systemic lymphoma (sPTCL-NOS) [8]. Other common extranodal sites are bone marrow, liver, spleen, lungs, and gastrointestinal tract [7–9].

Although liver biopsy was not performed, we suspected hepatic involvement of PTCL-NOS in this case, owing to acute liver dysfunction and improvement of elevated hepatobiliary enzymes after steroid administration. Therefore, obstruction of the biliary tract was suspected. At a single North American institution [10], it was reported that liver functional disorder occurs in only 10 out of 117 cases diagnosed as PTCL-NOS (approximately 9%).

In our case, uveitis and anterior chamber inflammation were found on ophthalmic examination. Though vitreous biopsy was not performed, we suspected Uveitis masquerade syndrome due to intraocular lymphoma. Most cases of Uveitis masquerade syndrome with lymphoma are a result of large-cell lymphoma, with few reports of intraocular lesions due to PTCL.

In our case, rapidly progressive renal failure was confirmed. On renal biopsy, we confirmed infiltration of peripheral T-cell lymphoma, not otherwise specified (PTCL-NOS) into the renal tissue.

Infiltration of T-cell lymphoma into the kidney is rare, and we did not find reports of infiltration of PTCL-NOS into the kidney in the current literature. There are also no reports on the characteristics of renal lymphoma caused by PTCL-NOS in the literature. This is a case of perivascular tubulointerstitial nephritis with the direct invasion of PTCL-NOS. Since we did not find any glomerular lesions or cast nephropathy, we think this case of primary or secondary PTCL-NOS, directly infiltrating into the tubulointerstitial tissue and causing rapidly progressive renal failure, is extremely rare.

In our case, a single steroid dose showed dramatic results with respect to renal, intraocular, hepatic, and cutaneous symptoms. Obrador GT, et al. reported a case of acute renal failure secondary to massive B-cell lymphomatous infiltration of the kidneys, which was rapidly reversed with high-dose steroid therapy before introduction of CHOP therapy, which is similar to our approach [11].

Steroid therapy in malignant lymphoma is believed to induce apoptosis in the tumor cells via a glucocorticoid receptor [12]. Therefore, steroid sensitivity of malignant lymphoma cells depends on the number of glucocorticoid receptor or variants of the receptor in cells. Resistance to steroid depends on abnormal expression of the glucocorticoid receptor, especially mutation of the glucocorticoid receptor gene [13–15]. In this case, we can infer that the malignant lymphoma cells had numerous glucocorticoid receptors.

The case reaffirms the importance of renal biopsy to diagnose acute kidney injury (AKI) of unknown cause, with systemic symptoms.

## Abbreviations

AITL: Angioimmunoblastic T-cell lymphoma
AKI: Acute kidney injury
ALCL: Anaplastic large-cell lymphoma
ALP: Alkaline phosphatase
ALT: Aalanine transaminase
AST: Aspartate aminotransferase
ATLL: Adult T-cell leukemia lymphoma
CHOP: Cyclophosphamide, doxorubicin, vincristine, and prednisone
Cr: Creatinine
CT: Computed tomography
FDG: Fluorodeoxyglucose.
HTLV-I: Human T-lymphotropic virus type I
IOP: Intraocular pressure
JCS: Japanese Coma Scale
pcptcl-NOS: Primary cutaneous disease
PTCL: Peripheral T-cell lymphoma
PTCL-NOS: Peripheral T-cell lymphoma, not otherwise specified
SpO_2_: Peripheral capillary oxygen saturation
sptcl-NOS: Systemic lymphoma
γ—GTP: γ-glutamyl transpeptidase
